# Predicting the Unpredictable: A Data-Driven Machine Learning Model for Emergency Department Waiting Room Surge Status

**DOI:** 10.1016/j.mcpdig.2026.100365

**Published:** 2026-04-27

**Authors:** Derick Jones, Laura Walker, Lindsey Asher, Gregory Davis, Brendan Carr, James Colletti

**Affiliations:** a Department of Emergency Medicine, Mayo Clinic, Rochester, MN; b Administration, Department of Emergency Medicine, Mayo Clinic, Rochester, MN

## Abstract

**Objective:**

To develop a machine learning algorithm to predict emergency department (ED) waiting room surge status (green, yellow, and red categories) 4, 8, and 12 hours in advance.

**Patients and Methods:**

We designed a retrospective cohort with prospective validation conducted between July 1, 2023, and April 30, 2025. A total of 154,956 encounters were analyzed; features included ED operational metrics and timestamps with 72-hour lagging data. Deep neural network and gradient boosted decision tree (XGBoost) models were trained to predict 3 predefined surge categories: green (<15 waiting patients; 48.8% of hours), yellow (15-30 waiting patients; 38.8% of the hours), and red (≥31 waiting patients; 12.3% of the hours).

**Results:**

The XGBoost model found strong predictive performance across all forecast horizons. Area under the curve (AUC) curves showed excellent discrimination between green and yellow levels, ranging from 0.87 to 0.91 AUC across all time horizons, demonstrating reliable differentiation between normal and moderate surge conditions. The model showed acceptable discrimination for red levels, with AUCs of 0.76 and 0.77, meeting commonly accepted thresholds for clinical forecasting tools, particularly given the difficulty of predicting rare, high-volume situations. The operational accuracy of 68% to 70% showed strong real-world multiclass operational forecasting over prolonged windows up to 12 hours in advance.

**Conclusion:**

Using operational metrics with timestamps, XGBoost models can differentiate between different levels of ED surge states with meaningful accuracy. This ability to forecast risk of high volumes provides a window of opportunity to proactively modify operations.

Emergency department (ED) crowding remains a persistent challenge, with substantial implications for patient outcomes, clinician well-being, and overall operational efficiency. Most health systems lack effective models to accurately forecast waiting room surge status and dynamically adjust capacity resulting in overcrowding and inefficient resource allocation.

Advances in artificial intelligence (AI) and time-series forecasting offer promising solutions to extend previous work on ED volume prediction. Machine learning (ML) algorithms trained on historical ED data can identify patterns and anticipate waiting room fluctuations, with greater lead time and precision. Although several studies have successfully forecasted patient arrivals using advanced techniques such as gradient boosting and recurrent networks, few have targeted waiting room census categories that integrate both arrival input and throughput pressures for operational actionability.

Previous ED forecasting studies have primarily focused on arrival volume prediction rather than operational surge states. Daily arrival forecasts commonly report mean absolute percentage errors (MAPE) between 5% and 10%,^1^, ^2^, ^3^ whereas hourly forecasts typically show MAPE ranging from 8% to 30%.^4^^,^^5^ Deep learning models such as long short-term memory networks frequently outperform classical time-series approaches for short horizons, achieving MAPE as low as 5% in selected settings,^2^ although performance gains are often incremental and dependent on feature engineering and forecast horizon.^4^^,^^6^

More recently, ML approaches incorporating queue-state variables and operational flow features have found the ability to forecast ED occupancy and waiting time prediction in advance, often outperforming rolling-average baselines.^7^, ^8^, ^9^ However, these studies generally evaluate continuous census prediction rather than discrete surge tiers aligned with operational response protocols.

Binary overcrowding prediction models often based on National Emergency Department Overcrowding Score, occupancy rate, or boarding thresholds typically report area under the curve (AUC) values between 0.80 and 0.90.^10^, ^11^, ^12^ Although some multivariable models achieve high internal discrimination,^13^ performance frequently degrades under external or cross-site testing, emphasizing the importance of local validation and recalibration.^14^

Importantly, despite the substantial body of ED prediction research, prospective real-time deployment remains uncommon. A recent scoping review concluded that many prediction models do not progress beyond retrospective validation, and implementation within live operational workflows is inconsistent.^15^ Only a limited number of single-center pilot systems have reported real-time forecasting integration into ED dashboards or clinical decision–support environments.^16^, ^17^, ^18^

Accurate surge prediction is critical for maintaining adequate staffing, preserving care quality, and preventing system strain. Machine learning–based forecasting models provide proactive planning and agile operational decisions.^19^^,^^20^ These tools can optimize staff allocation, reduce patient wait times, and improve department flow, ultimately enhancing both patient and provider experience.^19^ By continuously retraining new data, AI systems can maintain accuracy over time and support intelligent shift scheduling without increasing total staffing.^20^

Accordingly, a gap persists in the development of multiclass, operationally actionable surge prediction models that integrate arrival and throughput dynamics, forecast across clinically meaningful multihour horizons, and show prospective validation within a live operational environment.

## Problem Statement

Not only staffing an ED to meet routine patient volume but also handling unpredictable surges have become increasingly complex owing to rising patient acuity, prolonged boarding, and limited inpatient capacity. The challenge is to use prediction models to reduce staffing costs without negatively affecting throughput.^21^ There is increasing patient demand and lack of long-term dynamic planning for ED surge to provide timely patient care, leading to overcrowding and suboptimal resource allocation.^22^

Machine learning offers a dynamic approach by identifying temporal patterns in operational data. Although previous studies have improved arrival forecasting, few have developed multiclass waiting room surge prediction models that integrate both arrival and throughput pressures for direct operational use.^1^, ^2^, ^3^, ^4^, ^5^, ^6^ The ability to reliably predict surge status prospectively across clinically meaningful horizons (4-12 hours) remains limited, representing a key gap this study addresses.

### Objectives

The primary objective of this study was to develop and prospectively validate an ML model for predicting ED surge status (green, yellow, and red categories) at 4, 8, and 12 hours in advance using internal operational data. The model aimed to enable proactive staffing and resource allocation to mitigate crowding, reduce patient wait times, and improve operational efficiency.

## Patients And Methods

### Study Design and Setting

**Figure 1:**
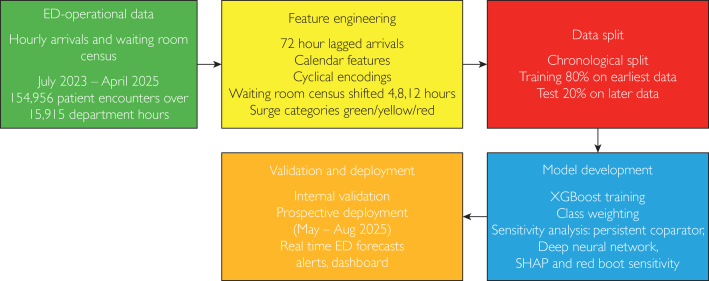
Flowchart illustrating the study pipeline from data extraction to prospective deployment. Raw emergency department (ED) operational data underwent feature engineering. Data were chronologically split. The gradient-boosted decision tree (XGBoost) model was developed and compared with a deep neural network and persistence baseline. Interpretability was assessed using Shapley additive explanations (SHAP) values. The final model was prospectively validated and deployed for real time hourly forecasting with automated alerts (May to August 2025).

We conducted a retrospective cohort study with prospective validation to develop, validate, and deploy ML model for predicting ED waiting room surge conditions 4, 8, and 12 hours in advance. This study adhered to transparent reporting of a multivariable prediction model for individual prognosis or diagnosis (TRIPOD)+AI guidelines for transparent reporting of multivariable prediction models developed using ML. The study took place at a large tertiary academic medical center in the Midwest United States, serving as a regional referral center, with an annual volume of approximately 85,000 visits. Figure 1 illustrates study workflow, data preparation chronological splitting, model development, internal validation, and prospective deployment.

### Study Population

All patients presenting to the ED between July 1, 2023, and April 30, 2025, were included in the analysis. During this period, 154,956 encounters spanning 15,915 hours were analyzed. No patient-level exclusion criteria were applied because the analysis used hourly operational metrics. The study was reviewed by the Mayo Clinic Institutional Review Board and deemed exempt due to the use of deidentified operational data.

### Data Sources

Data were extracted from the institution’s electronic medical record. Hourly arrival and roomed timestamps were used to calculate the following 2 operational metrics:

1. The number of patients in the waiting room at the start of each hour, and
2. The number of new arrivals per hour (count of patients arriving at the start of each hour).

Arrival to room intervals were aggregated hourly to calculate waiting room occupancy. For hours where there were no patients in the waiting room or there were no arrivals, 0 was imputed.

### Feature Engineering

Features were derived from hourly operational metrics to capture temporal patterns and arrival momentum (Table 1). Lagged features included hourly patient arrivals for the preceding 1 to 72 hours. Calendar features comprised year, quarter, month, week or month, day of week, hour of day, and a binary indicator for US federal holidays derived from the US Federal Holiday Calendar.

**Table 1:** Predictors Used in the Machine Learning Model

Category	Features	Description	Lagged	Encoding/notes
Target	target	Future waiting room census (shifted 4/8/12 h)	—	Numeric (float64)
Temporal (calendar)	year	Calendar year	No	Integer
Temporal (calendar)	quarter	Quarter of year	No	Integer
Temporal (calendar)	month	Month of year	No	Integer
Temporal (calendar)	week of month	Week within month	No	Integer
Temporal (calendar)	day of week	Day of week (0-6)	No	Integer
Temporal (calendar)	hour of day	Hour of day (0-23)	No	Integer
Temporal (calendar)	holiday	Binary US federal holiday indicator	No	Binary
Temporal (cyclic)	hour sin, hour cos	Sine/cosine of hour of day	No	Continuous (−1, 1); added in sensitivity analyses
Temporal (cyclic)	day of week sin, day of week cos	Sine/cosine of day of week	No	Continuous (−1, 1); added in sensitivity analyses
Temporal (cyclic)	month sin, month cos	Sine/cosine of month	No	Continuous (−1, 1); added in sensitivity analyses
Operational (lagged)	hourly arrivals lag 1 to hourly arrivals lag 72	Hourly patient arrivals lagged 1-72 h	Yes	Numeric (float64)
Surge status (derived)	Status	Categorical label (green/yellow/red) derived from target	—	Object (string); label encoded for modeling

In sensitivity analyses, cyclic sine and cosine transformations were applied to periodic temporal features (hour of day, day of week, and month) to better represent continuity. Both raw and cyclic encodings were retained, allowing the model to select the most predictive representation. No scaling was applied, because tree-based models are invariant to monotonic transformations.

### Outcome Definition

The primary outcome was the hourly ED waiting room surge status that is categorized into 3 operational levels based on waiting room census: green, less than 15 patients; yellow, 15-30 patients; and red, 31 patients or more. These thresholds correspond to institutional “traffic light” protocols for operational interventions and are derived from historical analysis of crowding impacts on flow and safety.

### Statistical Analyses and Modeling

Supervised ML models were trained to predict multiclass probabilities for surge status using the 1-vs-rest approach for evaluation metrics. Overall accuracy was the proportion of correctly predicted instances, and AUC was calculated 1-vs-rest.

The deployed model was a gradient-boosted decision tree (XGBoost) with default hyperparameters except for objective=“multi:softprob,” num class=3, and eval metric=“mlogloss.” Class imbalance was addressed via synthetic minority oversampling technique (SMOTE) applied exclusively to the training set.

In sensitivity analyses, SMOTE was replaced with balanced class weighting (inverse-frequency weights) to eliminate any potential temporal leakage concerns associated with synthetic oversampling in time-series data. A 3.0× boost was optionally applied to red class weights to prioritize surge detection. Cyclic sine/cosine encodings were added for periodic temporal features. A comparator deep neural network (feed-forward architecture with hidden layers of 128, 64, and 32 units; ReLU activation; light dropout [0.1]) was trained using balanced class weighting and standardized input features.

Interpretability was assessed using Shapley additive explanations (SHAP) values with TreeExplainer. A persistence baseline (current surge status as forecast) was evaluated as the primary naive comparator. Analyses were performed in Python 3.11 using XGBoost 1.7.6, scikit-learn 1.3.3, and SHAP. This study adhered to TRIPOD+AI guidelines.

### Ethics and Reporting Standards

This study was reviewed by the Mayo Clinic Institutional Review Board and deemed exempt from formal review owing to secondary analysis of deidentified operational data, with minimal risk to participants. The study involved aggregated hourly operational metrics without patient demographic characteristics, sex, gender, and race or ethnicity variables. These were not included because they were unrelated to study objectives. The study adhered to the TRIPOD+AI guidelines for transparent reporting of multivariable predictions models.

## Results

### Participant Characteristics and Surge Conditions

Between July 1, 2023, and April 30, 2025, there were 154,956 patient encounters over 15,915 department hours. The hourly distribution of waiting room surge conditions were as follows:

• Green (<15 patients): 48.8% of hours
• Yellow (15-30 patients): 38.8% of the hours
• Red (>31 patients): 12.3% of the hours.

### Model Performance

The deployed class-weighted XGBoost model (with SMOTE for imbalance) found strong predictive performance across all forecast horizons, achieving overall accuracy of 68% to 70% with 1-vs-rest AUCs of 0.87-0.88 (green), 0.8 to 0.91 (yellow), and 0.76 to 0.77 (red) on the internal test set. Predicted probabilities showed high confidence for green states and selective high confidence for red surges (Table 2). The distribution of predicted probabilities demonstrated clear separation between Green and Red surge states, with the Yellow class exhibiting broader overlap with both adjacent categories, reflecting its intermediate operational status (Supplemental Figure 1, available online at https://www.mcpdigitalhealth.org/).

**Table 2:** Model Performance Metrics Across Forecast Horizons

Analysis/model	Overall accuracy (%)	AUC green	AUC yellow	AUC red
4-h forecast	70	0.88	0.91	0.77
8-h forecast	68	0.87	0.89	0.76
12-h forecast	68	0.87	0.89	0.76

AUC, area under the curve.

### Sensitivity Analyses

**Figure 2:**
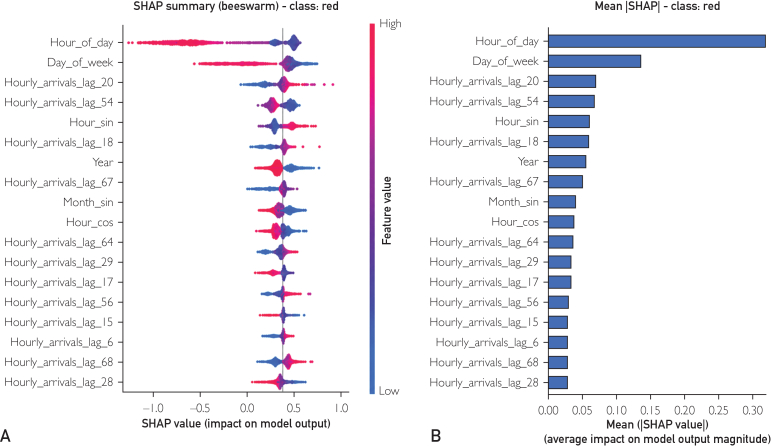
Global Shapley additive explanations (SHAP) analysis of the class-weighted gradient-boosted decision tree (XGBoost) model for the red surge class. (A) Beeswarm summary plot showing the distribution of SHAP values for each feature contributing to red predictions. Red dots indicate higher feature values pushing toward red; blue dots indicate lower values pushing away. Features are ranked by mean absolute SHAP value, with hour of day, day of week, and lagged arrivals (particularly lag 20 and lag 54) having the strongest impact. Cyclic encodings (hour sin) also rank highly, demonstrating their contribution to capturing diurnal patterns. (B) Bar plot of mean absolute SHAP values for the red class, confirming hour of day as the most influential feature, followed by day of week and multiple lagged arrival features. This analysis highlights the model’s reliance on temporal cycles and recent/multiday arrival momentum to identify high-risk surge conditions.

Several additional analyses were conducted using the 8-hour model to assess robustness, interpretability, and alternative approaches (Supplemental Table 1, available online at https://www.mcpdigitalhealth.org/). Incorporation of cyclic sine/cosine encodings for hour of day, day of week, and month improved SHAP interpretability of temporal patterns. A persistence baseline (current surge status as forecast) achieved only 30.1% accuracy, highlighting high temporal volatility. Replacing SMOTE with balanced class weighting yielded comparable performance. A 3.0× boost to red class weights increased red recall from 0.30 to 0.63, while maintaining overall accuracy approximately 63% to 68%. In post hoc comparator analyses, a deep neural network (balanced class weighting) achieved 69% overall accuracy with 1-vs-rest AUC values of 0.87 (green), 0.89 (yellow), and 0.78 (red), demonstrating performance comparable with the baseline XGBoost model. However, the neural network did not provide measurable improvement in high-surge (red) discrimination and offered reduced interpretability relative to gradient boosting, supporting the continued use of XGBoost for operational deployment. Global and local SHAP analyses (Figure 2 and Supplemental Figure 2, available online at https://www.mcpdigitalhealth.org/) consistently identified lagged arrivals and temporal features as dominant drivers.

### Model Interpretability Using SHAP Analysis

To provide insight into the decision-making process of the class-weighted XGBoost model (without SMOTE), SHAP were computed using TreeExplainer on the internal test set. Global feature importance (Figure 2) revealed that temporal patterns and lagged arrival momentum were the dominant drivers of surge predictions. The most influential features for the red class, ranked by mean absolute SHAP value, were as follows:

1. hour of day
2. day of week
3. hourly arrivals lag 20
4. hourly arrivals lag 54
5. hour sin
6. hourly arrivals lag 18
7. year
8. hourly arrivals lag 67
9. month sin
10. hour cos

The beeswarm feature summary plot (Figure 2) showed consistent directional effects: lower values of hour of day (morning predictions for evening surges) and lagged arrivals (particularly recent and multiday lags) strongly pushed predictions toward red, whereas higher values on hour of the day (evening predictions for early morning hours, low arrivals) favored green status. The bar plot (Figure 2) confirmed hour of day and day of week as the top contributors by overall impact.

Local explanations further illustrated clinical actionability. Supplemental Figure 2 presents a waterfall plot for a correctly predicted high confidence red surge (predicted probability=0.975). The prediction was driven predominantly by high arrivals 67 hours earlier (SHAP+1.1), cyclic day-of-week pattern (SHAP+0.5), early morning hour of day (SHAP+0.26), and multiple elevated lagged arrivals across 18 to 48 hours. These findings reflect compounding multiday operational pressure combined with diurnal and weekly cycle patterns, which are intuitively meaningful to ED leadership and directly support proactive resource activation. These global and local SHAP analyses satisfy explainable AI standards, enable clinician trust, and provide actionable insights into the drivers of predicted surges.

### Live Prospective Validation

The final XGBoost model was deployed in the ED’s live operational environment on Google Cloud Platform Vertex AI. Real-time data streams generated rolling 4-, 8-, and 12-hour surge forecasts and updated hourly to reflect the latest operational metrics. During approximately 2400 hours of prospective use (May 18 to August 26, 2025), model performance remained stable relative to internal validation.

**Figure 3:**
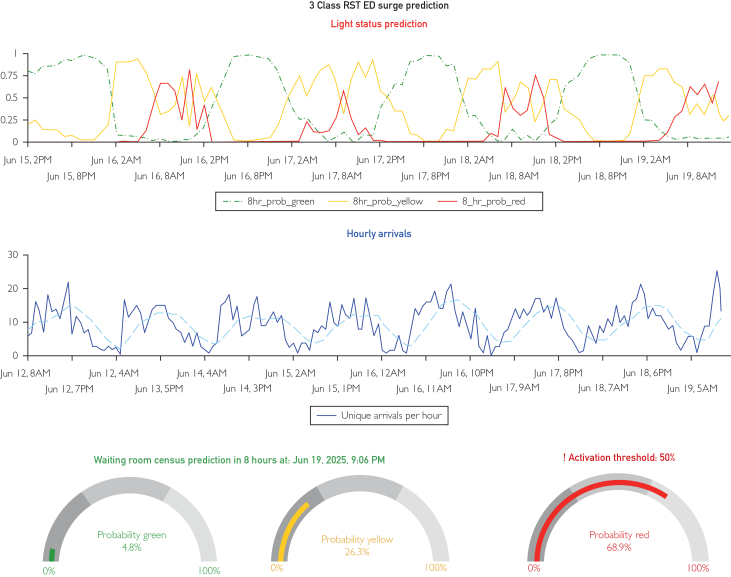
Live operational dashboard displaying real-time rolling surge forecasts with color-coded status and probability trends. E-mail alerts were triggered for red probabilities of >0.8, supporting leadership visibility and proactive resource planning. ED, emergency department. RST, Rochester ED

Alerts were triggered automatically via email notification to ED leadership when predicted probabilities for red status exceeded operational thresholds. Threshold selection focused primarily on red predictions to balance precision and recall, with initial testing at more than 0.5 probability and final implementation at more than 0.8 to prioritize specificity and minimize false alarms while preserving acceptable sensitivity (Supplemental Table 2, available online at https://www.mcpdigitalhealth.org/). Forecasts and probability trends were displayed on a dedicated live dashboard (Figure 3), providing continuous color-coded visibility (green, yellow, or red) across horizons for shift leaders and administrators.

Clinical and operational staff used the dashboard and e-mail alerts to support proactive decisions and resource reallocation during rising red probability trends. Although formal evaluation of downstream outcomes (eg, wait times and staffing efficiency) was not conducted, the system provided a decision-support tool with potential to enhance situational awareness and mitigate crowding.

## Discussion

This study extends previous ED forecasting research by targeting waiting room surge status as a direct integrator of arrival input and throughput pressure rather than arrivals alone. The deployed XGBoost model achieved robust performance (68%-70% accuracy; AUCs, 0.87-0.91 for green/yellow; 0.76-0.77 for red) across 4-, 8-, and 12-hour horizons, with stable prospective validation over 2400 hours.

Global and local SHAP analyses (Figure 2 and Supplemental Figure 2) revealed that temporal cycles (hour of day and day of week) and lagged arrival momentum (recent and multiday lags) were the dominant drivers. This provides transparent and clinically intuitive explanations for surge predictions. The persistence baseline performed poorly (30.1% accuracy), underscoring the high temporal volatility and the model’s added value in anticipating status changes (Supplemental Table 1, available online at https://www.mcpdigitalhealth.org/). The deep neural network achieved comparable performance but did not provide measurable improvement despite greater model complexity. Given its simplicity, stability, and superior interpretability, XGBoost was selected as the primary modeling approach.

Sensitivity analyses confirmed robustness: cyclic encodings enhanced temporal interpretability, and red weight class boosting increased recall modestly while maintaining overall accuracy. These findings support model tunability for operational preferences such as higher sensitivity vs specificity for alerts.

The prospective deployment provided real-time decision support via hourly forecasts with automated e-mail alerts (red probability>0.8) and a live dashboard (Figure 3). The potential operational benefits of forecasting are considerable and may allow administrative and clinical leaders to adjust staffing and allocate support services in a timely manner while preserving throughput and reducing costs.^21^^,^^22^ Most ED staffing models rely on static schedules developed weeks to months ahead, limiting flexibility when sudden demand arises.^22^ Although formal evaluation of downstream outcomes was not conducted, the system offers potential to improve situational awareness and facilitate proactive resource adjustments.

Beyond the ED, departments such as laboratory and radiology are directly affected by ED volume, and bottlenecks in these areas exacerbate crowding.^21^^,^^23^ Because ED arrivals directly influence inpatient occupancy, improved forecasting may strengthen hospital-level capacity planning.^24^

Importantly, this model predicts waiting room census rather than patient arrivals alone. Although related, waiting room census integrates both input and throughput pressures and provides a more comprehensive operational measure of crowding.^25^ To simplify implementation and support operational decision-making, waiting room census was categorized into discrete tiers based on established surge frameworks.^26^

Balancing precision and recall remains central to real-world deployment.^27^ At our site, model thresholds were optimized for specificity in detecting high-surge (red) conditions, accepting more false negatives to reduce unnecessary resource activation. This conservative approach not only promotes trust among clinical leaders but also allows calibration as familiarity with the model increases.

Future work should incorporate additional predictive features, including weather, hospital capacity, and community-level variables to enhance accuracy.^28^ Further experimentation with alternative class structures, such as simplifying to red vs nonred or expanding to 4 levels, may improve interpretability and operational performance.

### Limitations

This single-center study may limit generalizability. The model did not incorporate patient acuity, boarding status, or inpatient capacity, which can be key crowding drivers. Concept drift risk requires ongoing monitoring and retraining. External validation at other sites is needed. Rare extreme surges remain challenging to predict perfectly. Potential unintended consequences, such as alert fatigue or overreliance, warrant consideration.

## Conclusion

This study extends previous ED forecasting research by shifting focus from predicting patient arrivals to predicting real-time surge conditions in the ED and validating the results in a live operational environment. By enabling proactive, data informed staffing and resource allocation, surge prediction models have the potential to improve patient flow, reduce wait times, and enhance overall operational efficiency. Continued refinement, including integration of broader predictors and multicenter validation, will be essential to support widespread adoption across diverse emergency care environments.

## Potential Competing Interests

Dr Walker reports grants form Quai.md and leadership roles in Society for Academic Emergency Medicine and Academy of Women in Emergency Medicine. The other authors report no competing interests.

## Ethics Statement

This study was reviewed by the Mayo Clinic Institutional Review Board and deemed exempt from formal review owing to secondary analysis of deidentified operational data with minimal risk to participants. The study involved aggregated hourly operational metrics without patient demographic, sex, gender, and race or ethnicity variables. These were not included as they were unrelated to study objectives. The study adhered to the TRIPOD+AI guidelines for transparent reporting of multivariable predictions models.

## Declaration of Generative AI and AI-Assisted Technologies in the Writing Process

During the preparation of this work, the authors used Open AI Chat GPT in order to assist with drafting and refining sections of the manuscript and interpret statistical findings and to organize content into a manuscript structure. After using this tool, the authors reviewed and edited the content as needed and takes full responsibility for the content of the publication.
