# Supplementary material for: Predicting the Unpredictable: A Data-Driven Machine Learning Model for Emergency Department Waiting Room Surge Status
Source: Mayo Clin Proc Digit Health. 2026 Apr 27;4(2):100365. doi: 10.1016/j.mcpdig.2026.100365 (PMC13266022; doi:10.1016/j.mcpdig.2026.100365)
Supplement: Supplemental Material [file mmc1.docx]

**Supplementary Table 1:** Sensitivity Analysis and Comparators

| Analysis / Model | Overall Accuracy | AUC Green | AUC Yellow | AUC Red | Notes |
| --- | --- | --- | --- | --- | --- |
| Persistence baseline | 30.1 % | 0.43 | 0.45 | 0.47 | Current status as forecast |
| Deep Neural Network | 69 % | 0.87 | 0.89 | 0.78 | Similar performance to baseline model. |
| XGBoost class-weighted (no SMOTE) | 68 % | 0.87 | 0.90 | 0.76 | Eliminates leakage concern |
| XGBoost + 3.0× Red boost | 68 % | 0.87 | 0.89 | 0.76 | Red recall ↑ |
| XGBoost deployed (SMOTE) | **68 %** | **0.87** | **0.89** | **0.76** | **Primary model** |

**Supplementary Table 2.** Prospective Validation Performance Metrics for the XGBoost Model

| Class | Threshold | Support Positive | Support Negative | TP | FP | FN | TN | Prevalence | Precision | Recall | Specificity | NPV | F1 | Accuracy | Balanced Accuracy | Brier |
| --- | --- | --- | --- | --- | --- | --- | --- | --- | --- | --- | --- | --- | --- | --- | --- | --- |
| Green | 0.1 | 166 | 285 | 159 | 169 | 7 | 116 | 36.8% | 48.5% | 95.8% | 40.7% | 94.3% | 64.4% | 61.0% | 68.2% | 0.390244 |
| Green | 0.2 | 166 | 285 | 155 | 123 | 11 | 162 | 36.8% | 55.8% | 93.4% | 56.8% | 93.6% | 69.8% | 70.3% | 75.1% | 0.297118 |
| Green | 0.3 | 166 | 285 | 152 | 100 | 14 | 185 | 36.8% | 60.3% | 91.6% | 64.9% | 93.0% | 72.7% | 74.7% | 78.2% | 0.252772 |
| Green | 0.4 | 166 | 285 | 144 | 78 | 22 | 207 | 36.8% | 64.9% | 86.7% | 72.6% | 90.4% | 74.2% | 77.8% | 79.7% | 0.221729 |
| Green | 0.5 | 166 | 285 | 138 | 65 | 28 | 220 | 36.8% | 68.0% | 83.1% | 77.2% | 88.7% | 74.8% | 79.4% | 80.2% | 0.206208 |
| Green | 0.6 | 166 | 285 | 132 | 55 | 34 | 230 | 36.8% | 70.6% | 79.5% | 80.7% | 87.1% | 74.8% | 80.3% | 80.1% | 0.197339 |
| Green | 0.7 | 166 | 285 | 122 | 44 | 44 | 241 | 36.8% | 73.5% | 73.5% | 84.6% | 84.6% | 73.5% | 80.5% | 79.0% | 0.195122 |
| Green | 0.8 | 166 | 285 | 117 | 31 | 49 | 254 | 36.8% | 79.1% | 70.5% | 89.1% | 83.8% | 74.5% | 82.3% | 79.8% | 0.177384 |
| Green | 0.9 | 166 | 285 | 96 | 21 | 70 | 264 | 36.8% | 82.1% | 57.8% | 92.6% | 79.0% | 67.8% | 79.8% | 75.2% | 0.201774 |
| Yellow | 0.1 | 190 | 261 | 148 | 143 | 42 | 118 | 42.1% | 50.9% | 77.9% | 45.2% | 73.8% | 61.5% | 59.0% | 61.6% | 0.4102 |
| Yellow | 0.2 | 190 | 261 | 139 | 114 | 51 | 147 | 42.1% | 54.9% | 73.2% | 56.3% | 74.2% | 62.8% | 63.4% | 64.7% | 0.365854 |
| Yellow | 0.3 | 190 | 261 | 125 | 100 | 65 | 161 | 42.1% | 55.6% | 65.8% | 61.7% | 71.2% | 60.2% | 63.4% | 63.7% | 0.365854 |
| Yellow | 0.4 | 190 | 261 | 109 | 85 | 81 | 176 | 42.1% | 56.2% | 57.4% | 67.4% | 68.5% | 56.8% | 63.2% | 62.4% | 0.368071 |
| Yellow | 0.5 | 190 | 261 | 98 | 71 | 92 | 190 | 42.1% | 58.0% | 51.6% | 72.8% | 67.4% | 54.6% | 63.9% | 62.2% | 0.361419 |
| Yellow | 0.6 | 190 | 261 | 78 | 57 | 112 | 204 | 42.1% | 57.8% | 41.1% | 78.2% | 64.6% | 48.0% | 62.5% | 59.6% | 0.374723 |
| Yellow | 0.7 | 190 | 261 | 60 | 37 | 130 | 224 | 42.1% | 61.9% | 31.6% | 85.8% | 63.3% | 41.8% | 63.0% | 58.7% | 0.370288 |
| Yellow | 0.8 | 190 | 261 | 36 | 19 | 154 | 242 | 42.1% | 65.5% | 18.9% | 92.7% | 61.1% | 29.4% | 61.6% | 55.8% | 0.383592 |
| Yellow | 0.9 | 190 | 261 | 9 | 4 | 181 | 257 | 42.1% | 69.2% | 4.7% | 98.5% | 58.7% | 8.9% | 59.0% | 51.6% | 0.4102 |
| Red | 0.1 | 95 | 356 | 72 | 84 | 23 | 272 | 21.1% | 46.2% | 75.8% | 76.4% | 92.2% | 57.4% | 76.3% | 76.1% | 0.237251 |
| Red | 0.2 | 95 | 356 | 65 | 62 | 30 | 294 | 21.1% | 51.2% | 68.4% | 82.6% | 90.7% | 58.6% | 79.6% | 75.5% | 0.203991 |
| Red | 0.3 | 95 | 356 | 54 | 53 | 41 | 303 | 21.1% | 50.5% | 56.8% | 85.1% | 88.1% | 53.5% | 79.2% | 71.0% | 0.208426 |
| Red | 0.4 | 95 | 356 | 44 | 41 | 51 | 315 | 21.1% | 51.8% | 46.3% | 88.5% | 86.1% | 48.9% | 79.6% | 67.4% | 0.203991 |
| Red | 0.5 | 95 | 356 | 36 | 33 | 59 | 323 | 21.1% | 52.2% | 37.9% | 90.7% | 84.6% | 43.9% | 79.6% | 64.3% | 0.203991 |
| Red | 0.6 | 95 | 356 | 25 | 25 | 70 | 331 | 21.1% | 50.0% | 26.3% | 93.0% | 82.5% | 34.5% | 78.9% | 59.6% | 0.210643 |
| Red | 0.7 | 95 | 356 | 22 | 13 | 73 | 343 | 21.1% | 62.9% | 23.2% | 96.3% | 82.5% | 33.8% | 80.9% | 59.8% | 0.190687 |
| Red | 0.8 | 95 | 356 | 13 | 7 | 82 | 349 | 21.1% | 65.0% | 13.7% | 98.0% | 81.0% | 22.6% | 80.3% | 55.9% | 0.197339 |
| Red | 0.9 | 95 | 356 | 4 | 2 | 91 | 354 | 21.1% | 66.7% | 4.2% | 99.4% | 79.6% | 7.9% | 79.4% | 51.8% | 0.206208 |

**Supplementary Figure 1:** Probability Distributions and Threshold Based Partitioning

**
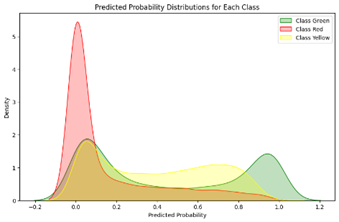
**

Predicted probabilities for the true class exhibited distinct patterns that support operational threshold-based alerting (Figure 4). The true Green instances showed a strong peak near probability 1, with a smaller peak near 0 indicating high model confidence for normal flow hours. True yellow instances were broadly distributed (0.3–0.8), reflecting overlap between normal and moderate conditions. True Red probabilities were right-skewed (<0.1) but with a noticeable small high-confidence tail approaching 1. This separation demonstrates the model’s ability to selectively identify rare surge events with high certainty, enabling calibrated probability thresholds to trigger alerts while minimizing false positives.

Note: Small density values below 0 and above 1 are artifacts of kernel density estimation smoothing and do not reflect actual model outputs, which are constrained to [0,1] by the softmax function.

**Supplementary Figure 2:** Local SHAP explanation for a high-confidence Red surge prediction in the class-weighted XGBoost model.

**
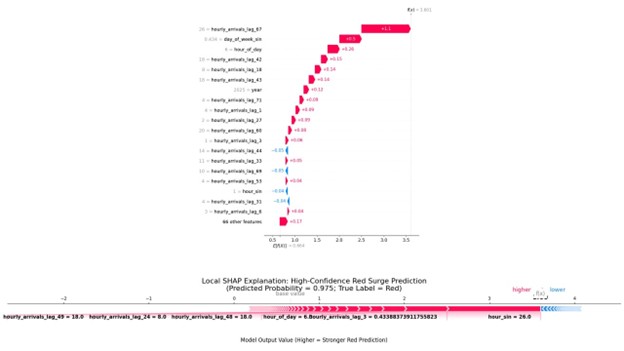
**

(A) Waterfall plot showing the additive SHAP contributions for a correctly predicted Red instance. The base value (expected model output = 0.664) is driven to a strong Red prediction (f(x) = 3.601) primarily by high arrivals 67 hours prior (SHAP +1.1), cyclic day-of-week pattern indicating a high-volume day (SHAP +0.5), and multiple recent lagged high-arrival hours. (B) Force plot for the same instance, visualizing the cumulative push from base value to final prediction. Red bars indicate features increasing surge risk; blue bars decrease risk. This example illustrates how the model integrates multi-day arrival momentum with temporal cycles to identify severe crowding.
